# Closing the box

**DOI:** 10.1080/17588928.2025.2537960

**Published:** 2025-07-31

**Authors:** Thomas Parr, Giovanni Pezzulo, Karl Friston

**Affiliations:** a Nuffield Department of Clinical Neurosciences, University of Oxford, UK; b Institute of Cognitive Sciences and Technologies, National Research Council, Rome, Italy; c Queen Square Institute of Neurology, University College London, UK

**Keywords:** Memory, attention, transformers, language, prediction

## Abstract

We were grateful for the level of engagement and insight from the commentaries on our discussion article, and for the opportunity to pick up on some of the common themes in what follows. Several commentaries focused upon the degeneracy in the relationship in how an internal model – of the sort that might be used either by an AI system or by our brains – might be formulated and the way in which this degeneracy might be resolved. Further themes were the role of model width as opposed to depth, the phenomenology of non-Markovian time, and a useful reminder that linguistic communication is necessarily a multi-agent, collective, endeavor.

## Same problem, different solution?

The phrase ‘closing the box’ in the title is a reference to a procedure used in graphical models, specifically Forney factor graphs (Loeliger et al., 2007), to represent the operation of marginalization. This is analogous to the computation of a partition function for a physicist (Landau & Lifshitz, 1980) or to the computation of model evidence for a statistician (MacKay, 1991). Curiously, it is a procedure that can be interpreted both as the transformation of a model from one form to another, equivalent, form, and as the process of forming inferences about the variables in that model by message passing. The reason this is relevant is that several of the commentaries – associated with our discussion paper – highlighted, in different ways, the insight that we might not have to choose between auto-regressive models and deep temporal models – the two may be entirely equivalent. Perhaps it is just the inferences used to solve them that differ.

This has relevance for the hardware (Muir & Sheik, 2025) or wetware (Isomura et al., 2023; Kagan et al., 2022) used to solve such problems, as solutions based upon distributed sparse message passing might be very well suited for the sorts of synaptic communication in biological brains (Parr et al., 2019). In contrast, the dense connectivity required prior to the application of an attention operator requires a different sort of computational architecture – or might rely upon densely connected (e.g., cortico-subcortical loop) subnetworks within the brain (Granier & Senn, 2025). The process theories (Friston et al., 2017) describing how a physical system might perform inference will differ based upon the system.

**Figure 1:**
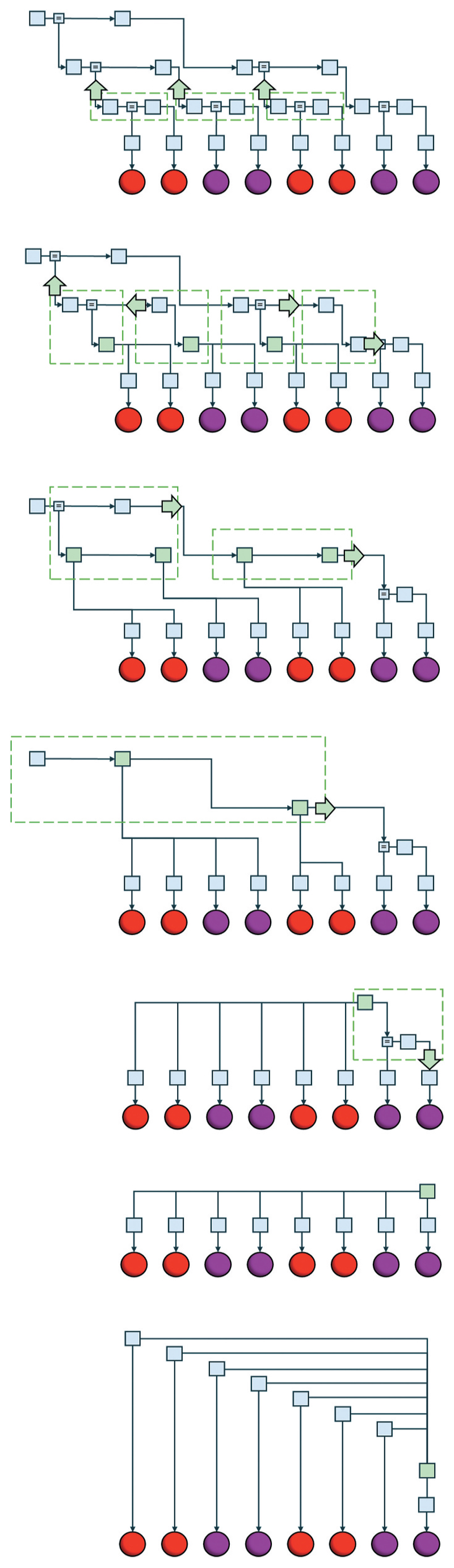
From deep temporal models to autoregression. This figure illustrates, through successive box closures, the transformation of a deep temporal (upper graph) to an autoregressive (lower graph) model architecture. Please see (Parr et al., this issue) for details of the graphical formalism. At each step, the factors outlined by the dashed green boxes are combined (by marginalizing over all variables on the internal edges) to give the green factors in the diagram below. The green arrows offer an interpretation of this process in terms of recursive message passing. The final graph, in which the previous values become part of the conditioning set for the green factor, depends upon renormalizing the graph such that the variable on the lower vertical edge of the green factor sums (or integrates) to one. This occurs implicitly when passing messages that originate from previous observations

Figure 1 highlights the relationship between message passing and marginalisation by using successive marginalisations (‘box closures’) to reformulate a deep temporal model as an autoregressive process. Boxes drawn around groups of factors indicate the summation or integration over all variables within those boxes, leading to the merging of multiple factor nodes in the following graph. This is the same procedure used to compute messages in sum—or integral—product Bayesian message passing schemes (Mooij & Kappen, 2007). This figure reinforces the points made by Singh et al. (this issue) and by (Sajid & Medrano, this issue) in showing, graphically, the equivalence between the two model architectures. One could argue that this means our brains use both deep temporal and autoregressive models, with the latter obtained in the final stages of synaptic message passing used to solve the former. This is an entirely reasonable interpretation. To unpack this further, particularly in relation to neurobiology, it will be useful to address questions of depth and width in generative models as raised by some of the other commentaries.

## Depth or width?

**Figure 2:**
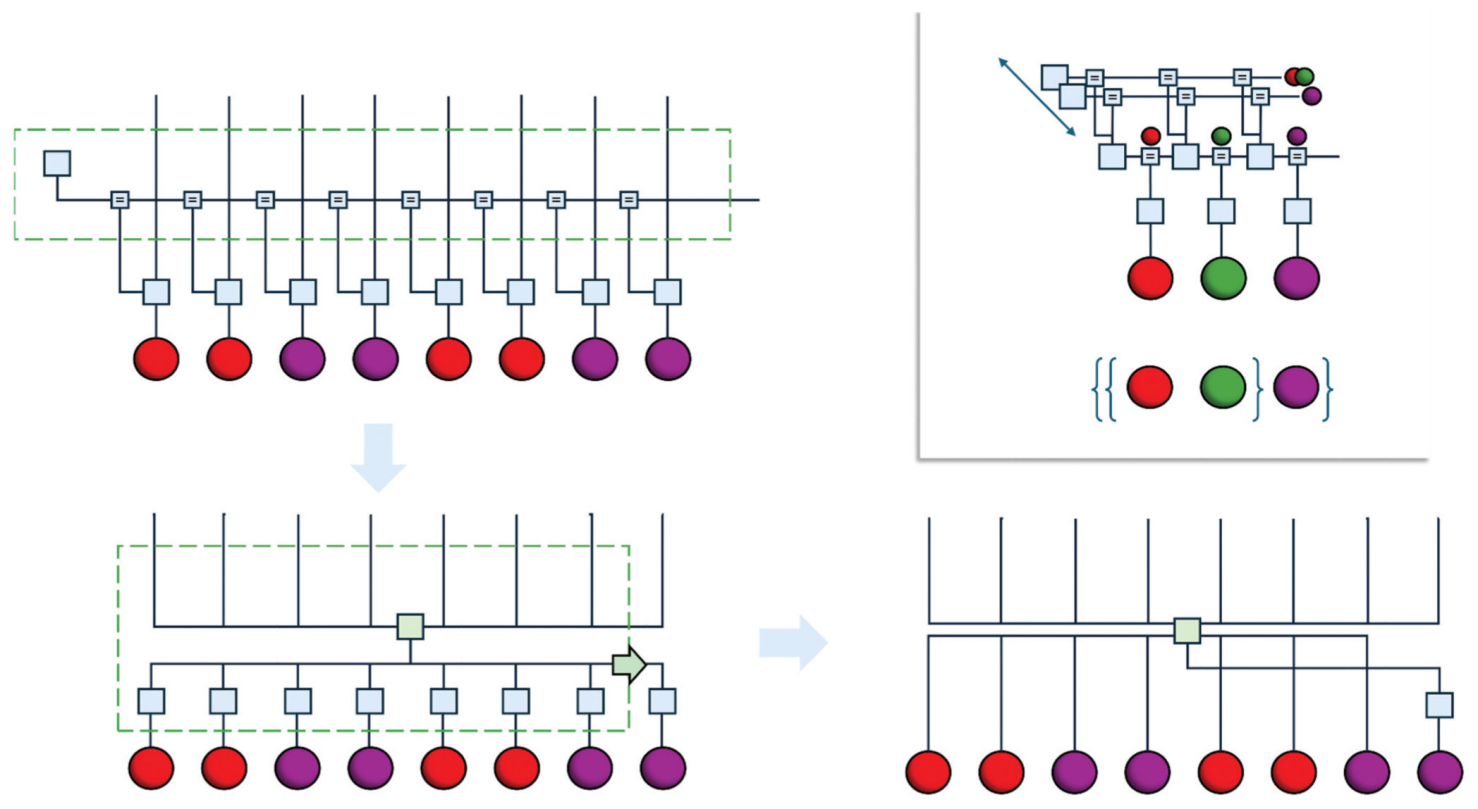
Thinking laterally. This figure illustrates the importance of lateral, or horizontal connectivity in relation to some of the ideas in the commentaries. The graphics here illustrate two distinct points that both relate to lateral connectivity. The first point is illustrated in the upper right plot that shows the form of a deep temporal model whose lower level deals with the ordering of symbols, while the upper level factorizes these into temporally invariant (at least, over a short timescale) syntactic groups, replicating syntactic trees of the sort discussed by Murphy (this issue). The second point is illustrated by the sequence of graphs from the upper left to lower right that show the development of a kind of highly lateralized expression characteristic in models (Gaussian processes being a common example) expressed in terms of kernels. The edges coming into each diagram from the top are the independent variables (e.g., pixel location) paired with the dependent variables (e.g., pixel intensity) aligned vertically. By marginalizing out the parameters mediating the mapping from one to the other, we arrive at an interpolative function that predicts the value of a dependent variable given its associated independent variable and previously observed pairs. This interpolation depends upon a metric space—a key element of Kiefer’s (this issue) commentary.

One could see the relationship above as analogous to that in which generative models based upon Gaussian processes can be re-expressed in terms of kernel operations (Rasmussen, 2004). Kernel methods predict datapoints based upon some function of previously measured datapoints, rather than from posterior inferences of hidden states. Figure 2 illustrates this idea for a model that predicts data given some explanatory variables. The upper left graph might be consistent with a simple linear model, with the edges coming in from the top representing independent variables (i.e., regressors) whose impact on measured data depends upon some common set of parameters (i.e., regression coefficients) shown along the horizontal line. An alternative expression of the same model is shown in the lower right, following box closures. Here, we have a factor that predicts the rightmost datapoint given all previous pairs of independent and dependent variables and the independent variable associated with the point to be predicted. Effectively, this is a kind of interpolation – which some would argue underwrites the power of all deep learning approaches (Belkin, 2021) – given some underlying metric space.

This is an important point of contact with the thoughts (Kiefer, this issue) offers on metric spaces. Kiefer’s commentary is a response to the question we raised concerning the apparently categorical structure of language compared to the metric embeddings used in large language models. In Gaussian processes, interpolation is possible because one expects that small variations in the value of an independent variable will cause only small variations in the value of the dependent variable. However, metric relationships are less obvious when variables are categorical, like words. As Kiefer points out, categorical entities inherit an implicit metric structure from the dependencies in a generative model. For instance, sparse transition probabilities taking us from one point in time to the next impose topological constraints. It may be that to get from one state of the world to another, we must pass through an intermediate state. The relative plausibility of different paths through a categorical state space gives that space a measure of ‘nearness’—i.e., a metric. Perhaps it is this metric that is implicitly captured by the token embeddings on which large language models rely.

Bringing this back to neurobiology, this speaks to subtle differences in the ways in which neuronal networks might be organized. The equivalence between (at least some) autoregressive (or kernel-based) and deep temporal models raises some interesting hypotheses. If our brains solve deep temporal models directly, we might anticipate a hierarchical organization, with relatively sparse synaptic communication between regions representing different timescales. However, if they solve autoregressive models in which some variables have been marginalized out, we might anticipate a flatter organizational structure with dense lateral connectivity within a given hierarchical level. Interestingly, there is evidence for both patterns of organization in biology (Felleman & Van Essen, 1991; Kiebel et al., 2008; Stettler et al., 2002; Zeki & Shipp, 1988), a prominent example being the use of neural field theories – that explicitly incorporate spatiotemporal connectivity kernels – to explain the spread of activity across neuronal populations (Coombes, 2005). This has particular relevance for the commentary by (Queißer et al., this issue), who highlight the importance of lateral connectivity, and argue convincingly that features of hierarchical processing can sometimes be replicated by shallow – but highly factorized – systems, in which different factors mutually contextualize one another. In other words, model width may be as important as (perhaps more important than) depth.

(Murphy, this issue) provides a useful illustrative example, by emphasizing factorization in language of the sort that might underwrite syntactic tree diagrams. The core idea here is that having temporal hierarchy is not enough. Once we have hierarchical levels that represent variables that are slowly changing – such that they appear constant during expression of a simple phrase – syntactic structure depends upon a careful accounting of the factorial structure at that timescale. The upper right panel of Figure 2 highlights how this might work in a model of the deep temporal sort, using colors in place of the symbols in the commentary. The horizontal factorization occurs at a higher, and therefore slower, level than the faster sequence below. The ordering into the sequence appropriate for a given ‘language’ is mediated by the messages passed to the level below. A treelike structure is represented by representing the combination of the red and green balls as a single factor, with the purple ball a factor of its own, replicating the grouping in the syntactic notation shown below. Further hierarchical levels, and their factorizations, might further decompose this tree.

## Time, agency, and communication

(Albarracin & Sakthivadivel, this issue) brings things back to our original premise – that one of the core challenges we face in characterizing complex language-like sequences is that they are highly non-Markovian and require models that incorporate long-range dependencies in time. If this were not the case, there would be no need for memory, as the present would tell us everything about the future without needing to recall past events. Albarracin and Sakthivadivel link this to concepts of consciousness and temporal depth, and to the non-Markovian explanations we use to account for the behavior of other creatures more generally. In short, when the observations we make of another creature are non-Markovian, we call upon the concepts of plans or policies that creature may have to explain this.

These themes of temporal extension, agency, and observation of others foregrounds the commentary by Taniguchi (this issue), who reminds us that language is ultimately a collective endeavor. One of the things that makes neurobiology so exciting is that to understand the nervous system, one must understand the world with which it interacts. When that world includes other creatures with nervous systems, capable of speech, then the problem of representing sequences of words cannot be dissociated from the communicative intent of speech and the use of language both to achieve preferred states and to seek answers to questions to resolve uncertainty about our environment and the entities that inhabit it. Perhaps it is the latter that necessitates linguistic internal models that are minimally ambiguous – and might explain why (Equation 9 of our discussion article (Parr et al., this issue)) the objectives for active learning (Friston et al., 2024) and training of autoregressive models appear so similar.

## Conclusion

In addition to the technical meaning outlined above, the phrase ‘closing the box’ might be interpreted in terms of the differing agendas of scientific and engineering disciplines. It is relatively common to hear deep learning architectures described as ‘black box’ systems – meaning they are mechanistically opaque. This is not necessarily problematic if one seeks to produce a specific functional outcome. However, scientific investigation depends upon trying to ‘open the box’ and articulate transparent accounts of what goes on inside to cause those functional outcomes. Transformers appear capable of (at least) approximating human linguistic behavior. Perhaps they offer an opportunity to open a box to better define the problem our brains solve when we converse.

Identical issues emerge in the identification of generative models in complex system modeling. In brief, the cause-effect structure of a dynamical system is generally parameterized in terms of a state-space model. For instance, the parameters might include the synaptic efficacies in a neural mass model for functional imaging (or electrophysiological) timeseries data. For any given parameterization, there exists a summary of the input–output relationships in terms of (Volterra) kernels, via the Fliess fundamental formula^1^ (Fliess, 1981). Volterra series can be thought of as analogous to a polynomial (Taylor) series expansion but in place of sums of polynomials, the terms of the expansion are convolutions of each Volterra kernel with previous measurements. The Volterra kernels can always be expressed in terms of a nonlinear autoregressive process that, effectively, maps data to data, without reference to latent states. Crucially, although one can always derive the kernels from the parameters of the generative model, one cannot derive the parameters from the kernels: see Table 1 in (Friston et al., 2014).

On this view, transformers learn the kernels, not the parameters of a generative model. In this sense, a closed-box architecture remains a black-box from the perspective of the generative model. This is problematic because one cannot place priors over the model parameters. In turn, this precludes Bayesian model selection and the ability to test hypotheses about different architectures (but perhaps allows one to test hypotheses about different kernels). The distinction between generative and autoregressive modeling underwrites some fundamental distinctions in neuroscience, e.g., effective *versus* functional connectivity, dynamic causal modeling *versus* Granger causality, mechanistic *versus* descriptive, and so on. In short, the way in which we close our boxes matters.

## Footnotes

1 Technically, the Fliess fundamental formula means that any transformer architecture can be expressed as a functional Taylor expansion and thereby characterized in terms of (high order) Volterra kernels.
